# Correction to: Vitamin D regulates insulin and ameliorates apoptosis and oxidative stress in pancreatic tissues of rats with streptozotocin‑induced diabetes

**DOI:** 10.1007/s11356-022-22427-9

**Published:** 2022-08-06

**Authors:** Fatima El Zahra M. Fathi, Kadry M. Sadek, Asmaa F. Khafaga, Abd Elwahab A. Alsenosy, Hanan A. Ghoneim, Sahar F. Mahmoud, Mohamed M. Zeweil

**Affiliations:** 1 Department of Biochemistry, Faculty of Veterinary Medicine, Damanhur University, Damanhour, 22516 Egypt; 2 Department of Pathology, Faculty of Veterinary Medicine, Alexandria University, Edfina, 22758 Egypt; 3 Department of Physiology, Faculty of Veterinary Medicine, Damanhur University, Damanhour, 22516 Egypt; 4 Department of Histology and Cytology, Faculty of Veterinary Medicine, Damanhur University, Damanhour, 22516 Egypt

**Correction to: Environmental Science and Pollution Research**

**https://doi.org/10.1007/s11356-022-22064-2**

The correct affiliations are shown in this article.

The Original article has been corrected.

